# EGFR-TKIs versus taxanes agents in therapy for nonsmall-cell lung cancer patients: A PRISMA-compliant systematic review with meta-analysis and meta-regression

**DOI:** 10.1097/MD.0000000000005601

**Published:** 2016-12-16

**Authors:** Na An, Yingshi Zhang, Huibin Niu, Zuojing Li, Jiayi Cai, Qingchun Zhao, Qing Li

**Affiliations:** a School of Life Sciences and Biopharmaceutis, Shenyang Pharmaceutical University; b Department of Pharmacy, General Hospital of Shenyang Military Area Command; c School of Pharmacy; d School of Medical Apparatus and Instruments, Shenyang Pharmaceutical University, Shenyang, P.R. China.

**Keywords:** EGFR-TKIs, meta-analysis, meta-regression, randomized controlled trials, taxanes

## Abstract

**Background:**

Currently, the nonsmall-cell lung cancer (NSCLC) is a worldwide disease, which has very poor influence on life quality, whereas the therapeutic effects of drugs for it are not satisfactory. The aim of our PRISMA-compliant systematic review and meta-analysis was to compare the efficacy and safety of epidermal growth factor receptor tyrosine kinase inhibitors (EGFR-TKIs) with Taxanes in patients with lung tumors.

**Methods:**

We collected randomized controlled trials (RCTs) of EGFR-TKIs (gefitinib, erlotinib) versus Taxanes (docetaxel, paclitaxel) for the treatment of NSCLC by searching PubMed, EMbase, and the Cochrane library databases until April, 2016. The extracted data on progression-free survival (PFS), progression-free survival rate (PFSR), overall survival (OS), overall survival rate (OSR), objective response rate (ORR), disease control rate (DCR), quality of life (QoL), and adverse event rates (AEs) were pooled. Disease-relevant outcomes were evaluated using RevMan 5.3.5 software and STATA 13.0 software.

**Results:**

We systematically searched 26 RCTs involving 11,676 patients. The results showed that EGFR-TKIs could significantly prolong PFS (hazard ratio [HR] = 0.78, 95% confidence interval [CI]: 0.66–0.92) and PFSR (risk ratio [RR] = 2.10, 95% CI: 1.17–3.77), and improve ORR (RR = 1.62, 95% CI: 1.38–1.91) and QoL. EGFR-TKIs had similar therapeutic effects to taxanes with respect to OS (HR = 1.00, 95% CI: 0.95–1.05) and OSR (RR = 1.03, 95% CI: 0.94–1.14). Furthermore, there were no significant differences between them in DCR (RR = 0.95, 95% CI: 0.88–1.03). Finally, EGFR-TKIs were superior to taxanes in most of all grades or grade ≥3 AEs.

**Conclusion:**

In the efficacy and safety evaluation, EGFR-TKIs had an advantage in the treatment of NSCLC, especially for patients with EGFR mutation-positive. The project was prospectively registered with PROSPERO database of systematic reviews, with number CRD42016038700.

## Abstract 2

Supplemental Digital Content is available in the text

## 1 Introduction

Carcinoma of lungs have become the first killer among all cancers, and is the leading cause of cancer-related mortality across the world, with a 5-year survival of less than 15%.^[1,2]^ Nonsmall-cell lung cancer (NSCLC) accounts for nearly 80% to 85% among all cases of lung cancers, locally advanced NSCLC 25% to 30% of all cases and metastatic diseases 40% to 50% of all cases.^[3,4]^ In the last decade, the therapeutic method for these patients consisted of 1st-line chemotherapy that can significantly improve the curative effects, such as gemcitabine, taxanes combining with platinum, and pemetrexed. However, the response rates are modest and after standard 1st-line therapy, several patients relapse of the malady, hence patients with NSCLC demand 2nd-line chemotherapy after 1st-line chemotherapy.^[5]^

Currently, it is safe to say that NSCLC patients benefit from taxanes agents, such as paclitaxel and docetaxel, which can be seen as representative of the new generation of anticarcinogen with a unique mechanism: they play a role in the microtubule and tubulin system, combine with free tubulin and promote tubulin assembly into stable microtubules, and inhibit their depolymerization. Therefore, they prevent the division of a large number of cells, leading to cell death.^[6,7]^ Among paclitaxel plus carboplatin as 1st-line treatment in advanced NSCLC.^[8]^ Apart from paclitaxel, docetaxel is paclitaxel in the process of structural transformation synthesized paclitaxel derivatives, which has high bioavailability and small side effects. Docetaxel is approved as 1st-line therapy in combination with cisplatin, as single-agent 2nd-line therapy, or as single-agent maintenance therapy for patients with advanced NSCLC in numerous countries.^[9,10]^

To date, epidermal growth factor receptor tyrosine kinase inhibitors (EGFR-TKIs) as molecular targeted therapeutical drugs have aroused people's attention, therein gefitinib and erlotinib have secured approvals for the treatment of advanced NSCLC, especially for those with sensitizing EGFR mutations.^[11]^ Nevertheless, different mutations may result in different structural changes, thereby affecting subsequent clinical outcomes.^[12]^ EGFR-TKIs play a role in tumor suppression by blocking the signal transduction of tumor cells, including inhibition of tumor cell proliferation, acceleration of apoptosis, and antiangiogenesis.^[13]^ Compared with other traditional medicines, gefitinib and erlotinib can prolong progression-free survival (PFS) in EGFR mutation-positive patients, and can be administered easily (orally). A phase 2B open-label randomized controlled trial has shown that gefitinib exhibits good tolerability and antitumor activities in NSCLC.^[14]^

Based on these, we performed a meta-analysis and meta-regression to explore the efficacy and safety of these medications for NSCLC patients, which could dedicate to make evidence-based clinical decisions for the treatment of pulmonary cancer.

## 2 Method

This review was conducted according to the Preferred Reporting Items for Systematic Reviews and Meta-analyses (PRISMA) statement.^[15]^ The project was prospectively registered with PROSPERO database of systematic reviews, number CRD42016038700.^[16]^

### 2.1 Data sources and search strategy

We systematically searched 3 search engines: PubMed, EMbase, and the Cochrane library from inception to April 2016. The search strategy included keywords and MeSH terms related to therapy using EGFR-TKIs and taxanes. Clinical trials were in any languages with patients presenting with NSCLC (see details in Table S1). We also scrutinized the reference list of relevant publications for additional studies.

### 2.2 Inclusion criteria

The relevant literature was selected carefully had to meet the following 4 criteria: all patients were diagnosed with NSCLC; the treatment arm were given EGFR-TKIs for therapy; while the control arm were given taxanes for cure; measurable outcomes were reported; and randomized controlled trials (RCTs). Ethical approval was not necessary, because of meta-analysis is a type of secondary statistics study, not directly associated with the subjects.

### 2.3 Data extraction

Two investigators (AN and ZYS) read the references and extracted the data independently. If we had any disagreements, we asked the 3rd investigator (LQ or ZQC). Every eligible study included the following information: the first author, publication year, trial design, sample size, age, gender, performance status, clinical phase, EGFR status, disease status, intervention, outcome assessment time, the main outcomes, risk of bias, and Newcastle-Ottawa Scale (NOS) score. The main outcomes were as follows: PFS, progression-free survival rate (PFSR), overall survival (OS), overall survival rate (OSR), objective response rate (ORR), disease control rate (DCR), quality of life (QoL), and adverse events (AEs).

### 2.4 Study quality assessment

The Cochrane Collaboration tool was used to evaluate the quality of these studies.^[17]^ We assessed the following 7 items of risk of bias: random sequence (selection bias), allocation concealment (selection bias), blinding of participants and personnel (performance bias), blinding of outcome assessment (detection bias), incomplete outcome data (attrition bias), selective reporting (reporting bias), and other bias. Low risk, high risk, and unclear risk were classified in all studies. The NOS^[18,19]^ as recommended by the Cochrane Non-Randomized Studies Methods Working Group, our meta-analysis also used NOS to assess the quality of the included RCTs. NOS using the following criteria label as “yes,” or “no”: Is the case definition adequate? Representativeness of the cases? Selection of Controls? Definition of Controls? Comparability of cases and controls? (0–2) Ascertainment of exposure? (0–2) Same method of ascertainment for cases and controls? and nonresponse rate? We excluded some studies which score less than 5 score.

### 2.5 Statistical analysis

The hazard ratio (HR), risk ratio (RR), and odds ratio (OR) with 95% confidence interval (CI) were used in eligible study. If studies did not have significant heterogeneity (*P* > 0.10, *I*^2^ < 50%), fixed-effects model was calculated. By contrast, random-effects model was employed. Furthermore, using subgroup analysis for the possible causes of sources of heterogeneity factors. The results of the meta-regression with the *P* value less than 0.05 means that the factors could cause significant impact to overall. Funnel plot was produced to assess publication bias. All statistical analyses were conducted with Review Manager 5.3.5 statistical software (Cochrane Collaboration) and STATA 13.0 software (StataCorp, College Station).

## 3 Results

### 3.1 Article selection and risks of bias

**Figure 1:**
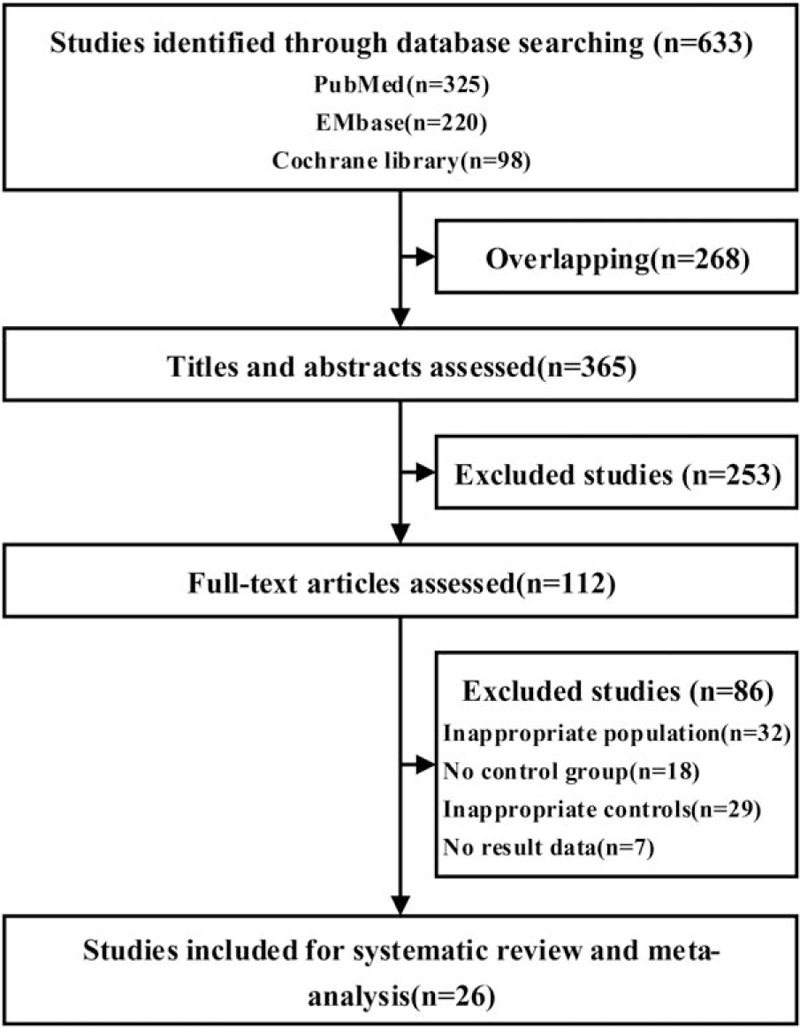
Flow of studies through the review process.

After searching the PubMed, EMbase, and the Cochrane library, we identified 633 articles, based on title and abstract screening, and obtained as full texts records. A total of 26 studies were included (Fig. 1).

We evaluated the risks of bias of all articles by the Cochrane Collaboration's tool and NOS scale, the required data can be evaluated as acceptable quality. The detail of quality assessment was shown in Table 1, Table S2 and Fig. S1.

**Table 1:**
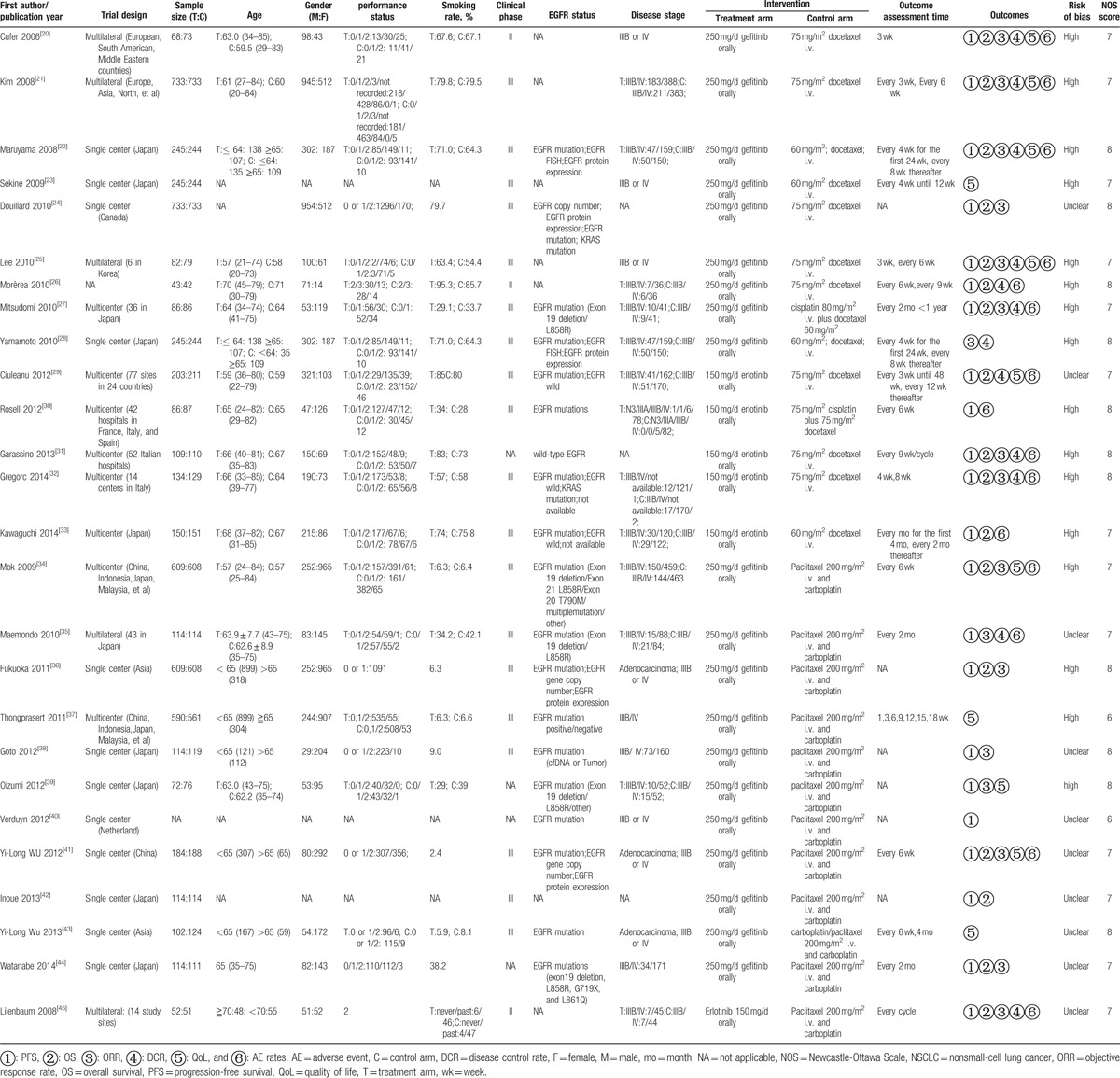
General condition sheet of included study.

### 3.2 Characteristics of included studies

The detailed characteristics of 26 studies were presented in Table 1. All the studies involved 11,676 patients, among which 5836 patients who received gefitinib/erlotinib were used as the treatment group and 5840 patients who received docetaxel/paclitaxel as the control group. Nine studies^[20–28]^ compared gefitinib versus docetaxel. Five studies^[29–33]^ compared erlotinib versus docetaxel. Eleven studies^[34–44]^ compared gefitinib versus paclitaxel. One studies^[45]^ compared erlotinib versus paclitaxel. Twenty-five studies^[20–26,28–45]^ were randomized. Nineteen studies^[22,24,27–41,43,44]^ included EGFR status, for example, EGFR mutation, EGFR wild-type, EGFR protein expression, and EGFR gene copy number. Taxanes combine with platinum and taxanes alone were used in 14 studies^[27,30,34–45]^ and 12 studies,^[20–26,28,29,31–33]^ respectively. Three studies^[20,26,45]^ were classified by phase II clinical trials, and 19 studies^[21–25,27–30,32–38,41–43]^ were classified by phase III. Thirteen studies^[20,21,25,27,29–35,37,45]^ were designed as multicenter and 12 studies^[22–24,28,36,38–44]^ were designed as single center.

### 3.3 Outcome evaluation and meta-analysis

#### 3.3.1 Progression-free survival (PFS), progression-free survival rate (PFSR)

**Figure 2:**
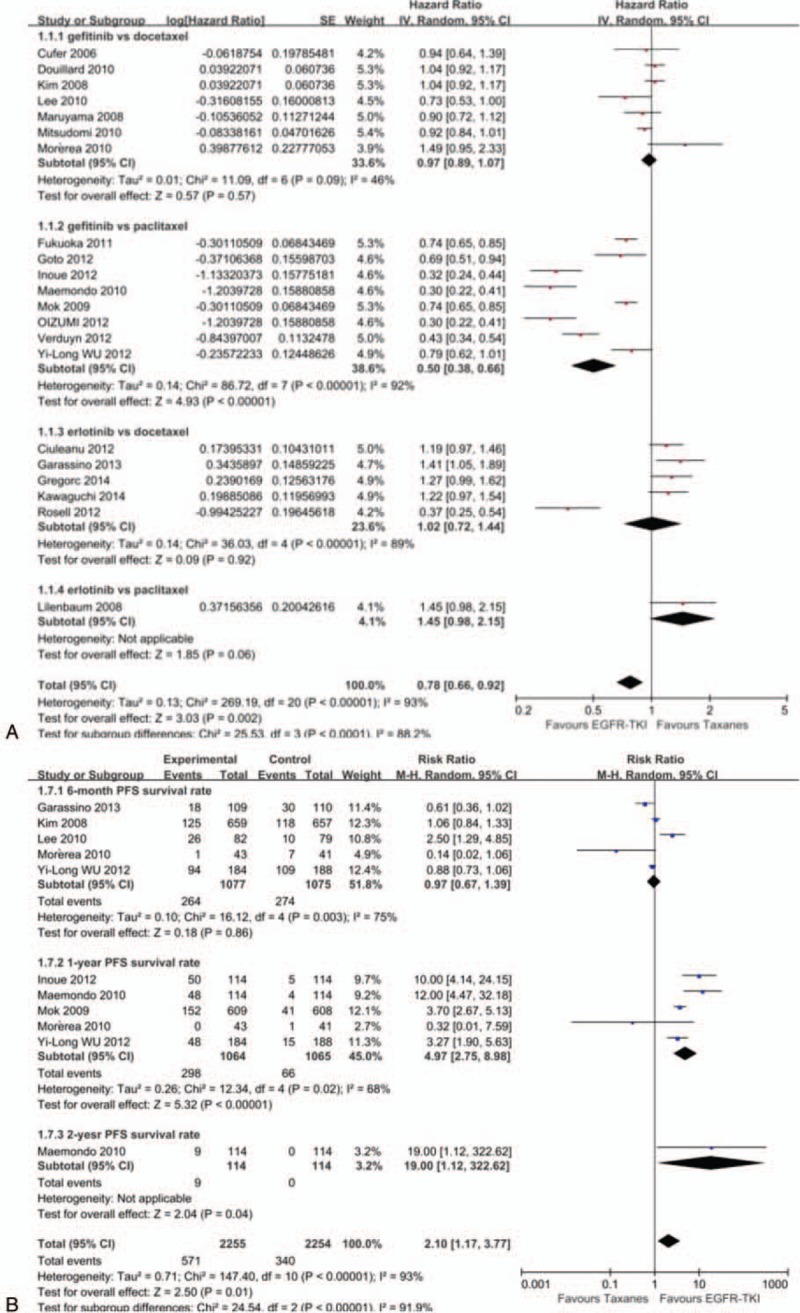
Forest plot of comparison for PFS (A) and PFSR (B) between EGFR-TKIs and taxanes in NSCLC. EGFR-TKI = epidermal growth factor receptor tyrosine kinase inhibitor, NSCLC = nonsmall-cell lung cancer, PFS = progression-free survival, PFSR = progression-free survival rate.

Twenty-one studies^[20–22,24–27,29–36,38–42,45]^ were finally included for analysis, which included 9096 patients, and 1 study^[44]^ was excluded due to irrelevant data. According to different drug types, the studies could be divided into 4 groups. There was significant heterogeneity between the included studies (*P* < 0.00001, *I*^2^ = 93%) and subgroup (*P* < 0.0001, *I*^2^ = 88.2%). Therefore, random-effect model was used for analysis. Comparing paclitaxel, gefitinib can significantly prolong PFS in patients (HR = 0.50, 95% CI: 0.38–0.66). There was no statistically significant difference in gefitinib versus docetaxel (HR = 0.97, 95% CI: 0.89–1.07), erlotinib versus docetaxel (HR = 1.02, 95% CI: 0.72–1.44), and erlotinib versus paclitaxel (HR = 1.45, 95% CI: 0.98–2.15). In general, the PFS was significantly longer in the EGFR-TKIs group than taxanes groups in patients with NSCLC (HR = 0.78, 95% CI: 0.66–0.92). Then, 5 studies^[20,24,25,33]^ reported 6-month PFSR (RR = 0.97, 95% CI: 0.67–1.39), no significant difference was detected between the 4 treatment arms in patients. Five studies^[25,27,28,33,34]^ reported 1-year PFSR (RR = 4.97, 95% CI: 2.75–8.98) and only 1 study^[28]^ reported 2-year PFSR (RR = 19, 95% CI: 1.12–322.62), we can know EGFR-TKIs can significantly prolong 1-year/2-year PFSR in patients. Overall, EGFR-TKIs were superior to taxanes in patients with NSCLC (RR = 2.10, 95% CI: 1.17–3.77) (Fig. 2).

#### 3.3.2 Overall survival (OS), overall survival rate (OSR)

**Figure 3:**
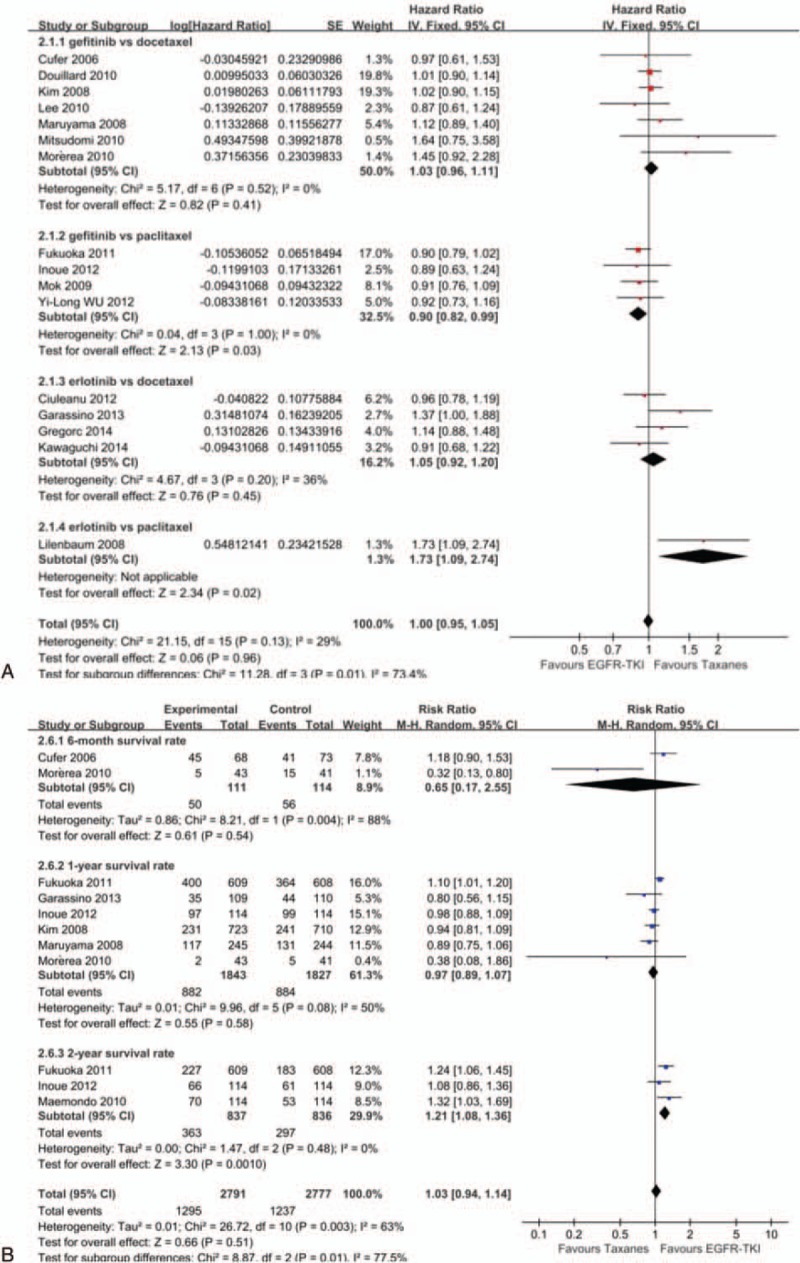
Forest plot of comparison for overall survival (A) and overall survival rate (B) between EGFR-TKIs and taxanes in NSCLC. EGFR-TKI = epidermal growth factor receptor tyrosine kinase inhibitor, NSCLC = nonsmall-cell lung cancer, OS = overall survival, OSR = overall survival rate.

Sixteen studies^[20–22,24–27,29,31–34,36,41,42,45]^ including 8539 patients were finally included for OS analysis. Because of not relevant data, 1 study^[44]^ was not included finally. No significant heterogeneity was presented in studies (*P* = 0.13, *I*^2^ = 29%). Therefore, we used fixed-effects model for analysis. Gefitinib produced longer OS than paclitaxel (HR = 0.90, 95% CI: 0.82–0.99). No significant differences were observed in gefitinib (HR = 1.03, 95% CI: 0.96–1.11) or erlotinib (HR = 1.05, 95% CI: 0.92–1.20) versus docetaxel. Erlotinib was inferior to paclitaxel in OS (HR = 1.73, 95% CI: 1.09–2.74). In summary, there was only a nonsignificant trend toward improved OS for the EGFR-TKIs over taxanes groups (HR = 1.00, 95% CI: 0.95–1.05). From Fig. 3B, we can get that EGFR-TKIs cannot significantly prolong 6-month/1-year OSR in patients (RR = 0.65, 95% CI: 0.17–2.55; RR = 0.97, 95% CI: 0.89–1.07), but it can significantly prolong 2-year OSR in patients (RR = 1.21, 95% CI:1.08–1.36). Overall, in terms of survival rate, EGFR-TKIs had equally therapy value to taxanes (RR = 1.03, 95% CI: 0.94–1.14) (Fig. 3).

#### 3.3.3 Objective response rate (ORR)

**Figure 4:**
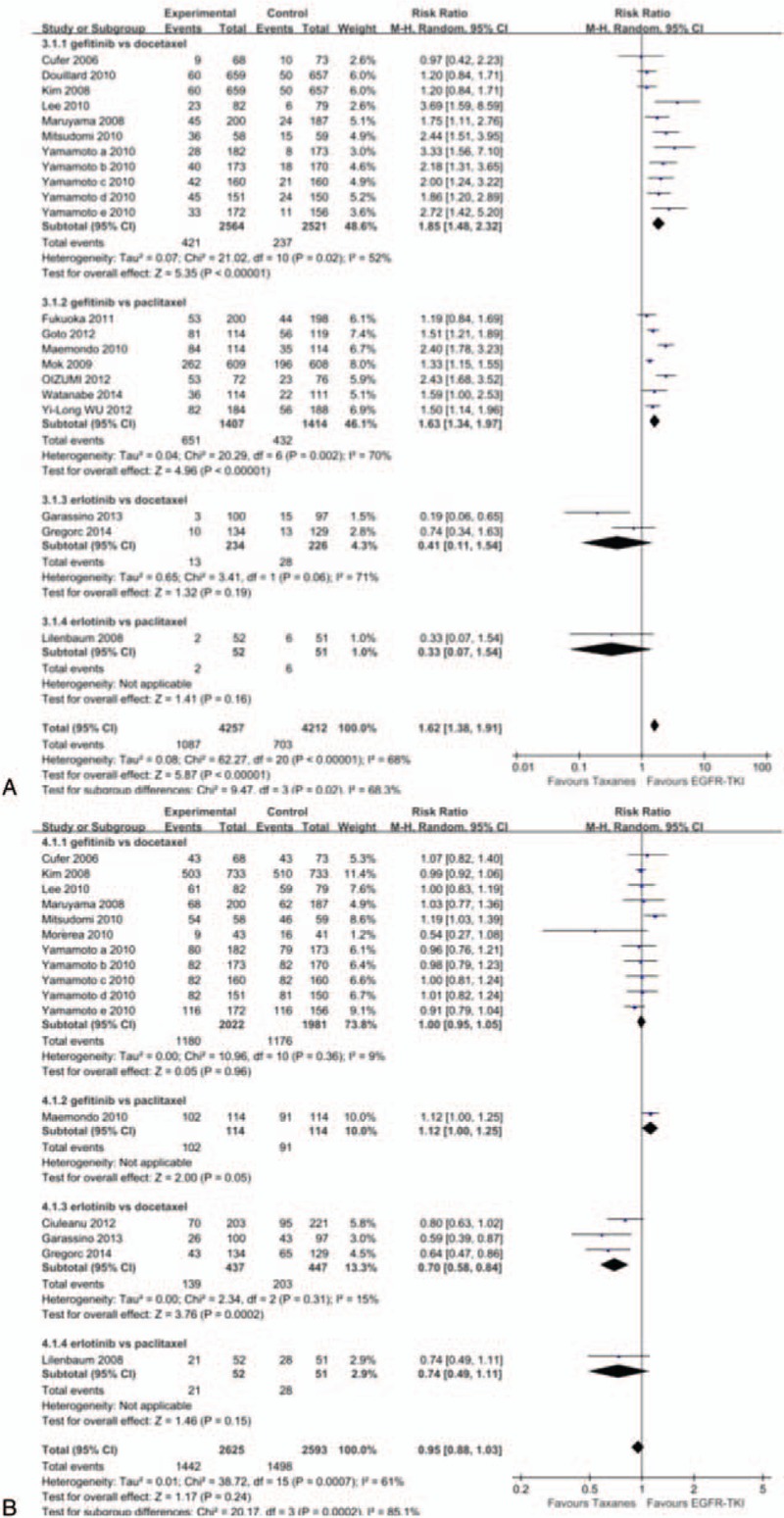
Forest plot of comparison for objective response rate (A) and disease control rate (B) between EGFR-TKIs and taxanes in NSCLC. EGFR-TKI = epidermal growth factor receptor tyrosine kinase inhibitor, NSCLC = nonsmall-cell lung cancer.

A total of 8469 patients were enrolled on these 17 studies^[20–22,24,25,27,28,31,32,34–36,38,39,41,44,45]^ (21 analyses) for ORR analysis. Significant heterogeneity was existed in included studies (*P* < 0.00001, *I*^2^ = 68%). Whether docetaxel (RR = 1.85, 95% CI: 1.48–2.32) or paclitaxel (RR = 1.63, 95% CI: 1.34–1.97), gefitinib can improve patient's ORR. There was no significant difference between erlotinib versus docetaxel (RR = 0.41, 95% CI: 0.11–1.54) and erlotinib versus paclitaxel (RR = 0.33, 95% CI: 0.07–1.54). Overall, EGFR-TKIs produced higher ORR than Taxanes in patients with NSCLC (RR = 1.62, 95% CI: 1.38–1.91) (Fig. 4A).

#### 3.3.4 Disease control rate (DCR)

Twelve studies^[20–22,25–29,31,32,35,45]^ (16 analyses) were identified, covering a total of 5218 subjects for DCR analysis. Significant heterogeneity among studies (*P* = 0.0007, *I*^2^ = 61%). Except the result of erlotinib versus paclitaxel (RR = 0.7, 95% CI: 0.58–0.84) indicated that paclitaxel can increase DCR, no significant differences were observed in additional three therapy groups (Fig. 4B).

#### 3.3.5 Quality of life (QoL)

**Figure 5:**
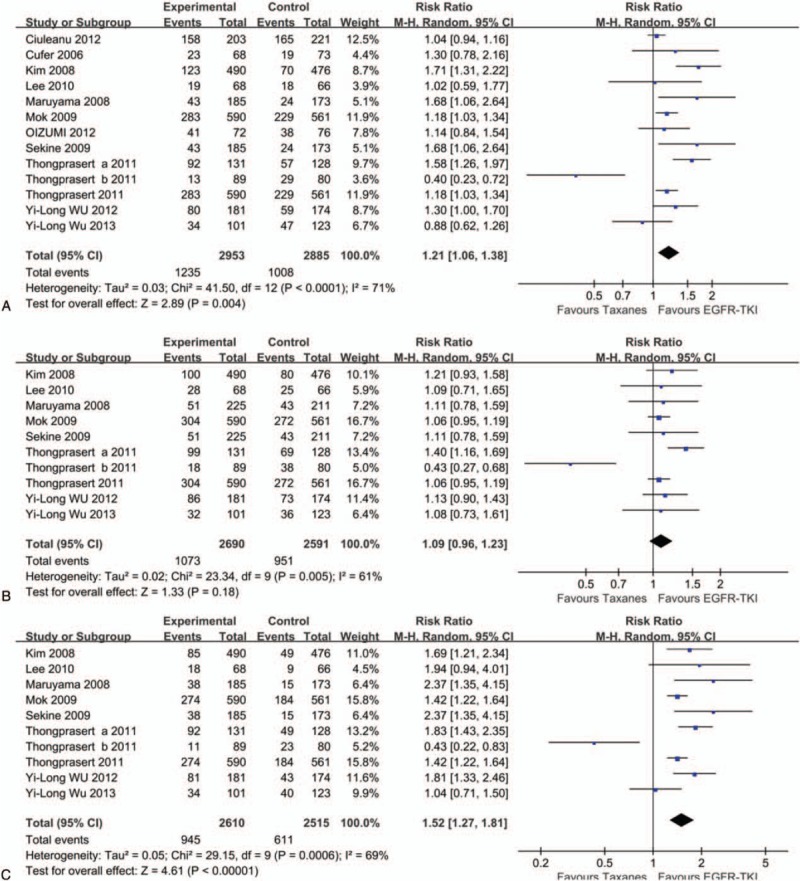
Forest plot of comparison for Functional Assessment of Cancer Therapy-Lung (A), Lung Cancer Subscale (B), and Trial Outcome Index (C) between EGFR-TKIs and taxanes in NSCLC. EGFR-TKI = epidermal growth factor receptor tyrosine kinase inhibitor, NSCLC = nonsmall-cell lung cancer.

We used Functional Assessment of Cancer Therapy-Lung (FACT-L), Trial Outcome Index (TOI), and Lung Cancer Subscale (LCS) to assess the QoL. The results of FACT-L analysis (RR = 1.21, 95% CI: 1.06–1.38), LCS analysis (RR = 1.09, 95% CI: 0.96–1.23), and TOI analysis (RR = 1.52, 95% CI: 1.27–1.81) showed that EGFR-TKIs group had better QoL than taxanes groups (Fig. 5).

#### 3.3.6 Adverse event rates (AEs)

The OR and 95% CI for common AEs were shown in Table 2. Comparing taxanes, EGFR-TKIs led to a lower rate in hematologic toxicity, alopecia, myalgia, pyrexia, and gastrointestinal reaction, except diarrhea (all grades OR = 1.92, 95% CI: 1.55–2.39; grade ≧3 OR = 1.70, 95% CI: 1.18–2.47). Meanwhile, rash was more common in the EGFR-TKIs groups (all grades OR = 4.62, 95% CI: 3.46–6.17; grade ≧3 OR = 4.60, 95% CI: 2.90–7.32). There was a similar incidence of constipation and pyrexia in grade ≧3.

**Table 2:**
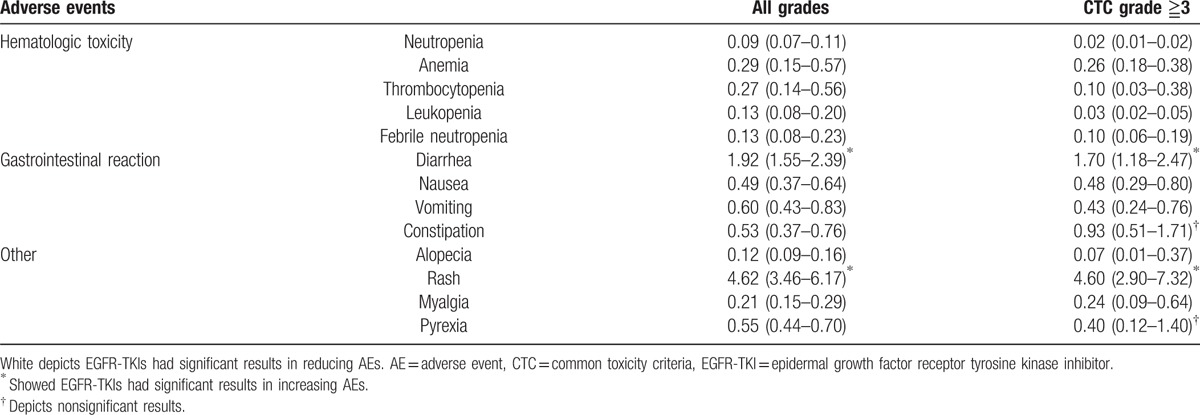
Summary of all AEs rate.

### 3.4 Subgroup analyses and meta-regression

Table 3  presented a summary of subgroup meta-analyses and meta-regression performed. EGFR status, platinum in control arm, clinical phase of trials, and trial design may have resulted in significant and substantial heterogeneity in our analysis; therefore, the study can be divided into 4 subgroups.

**Table 3:**
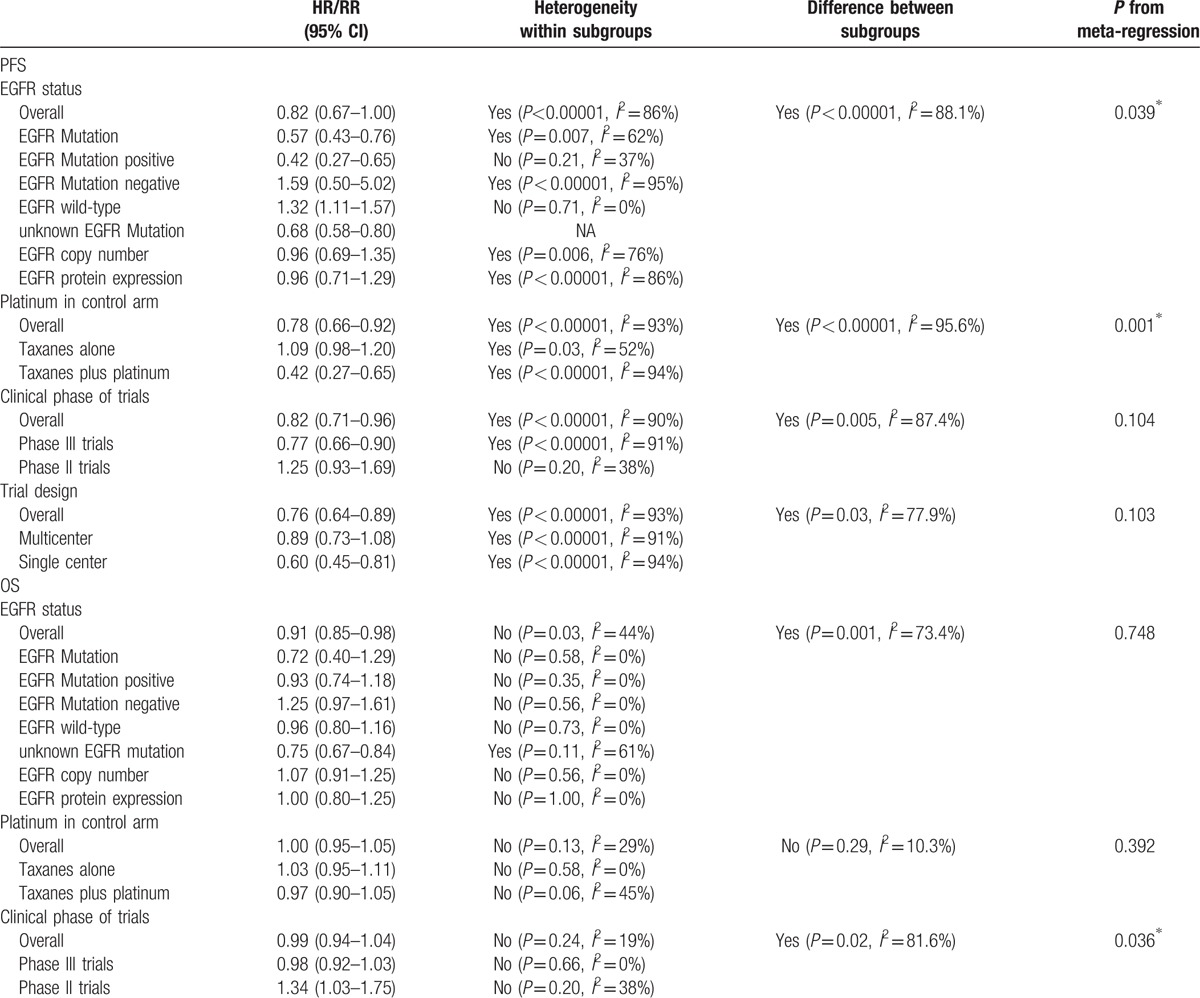
Summary of subgroup meta-analyses and meta-regression.

**Table 3 (Continued):**
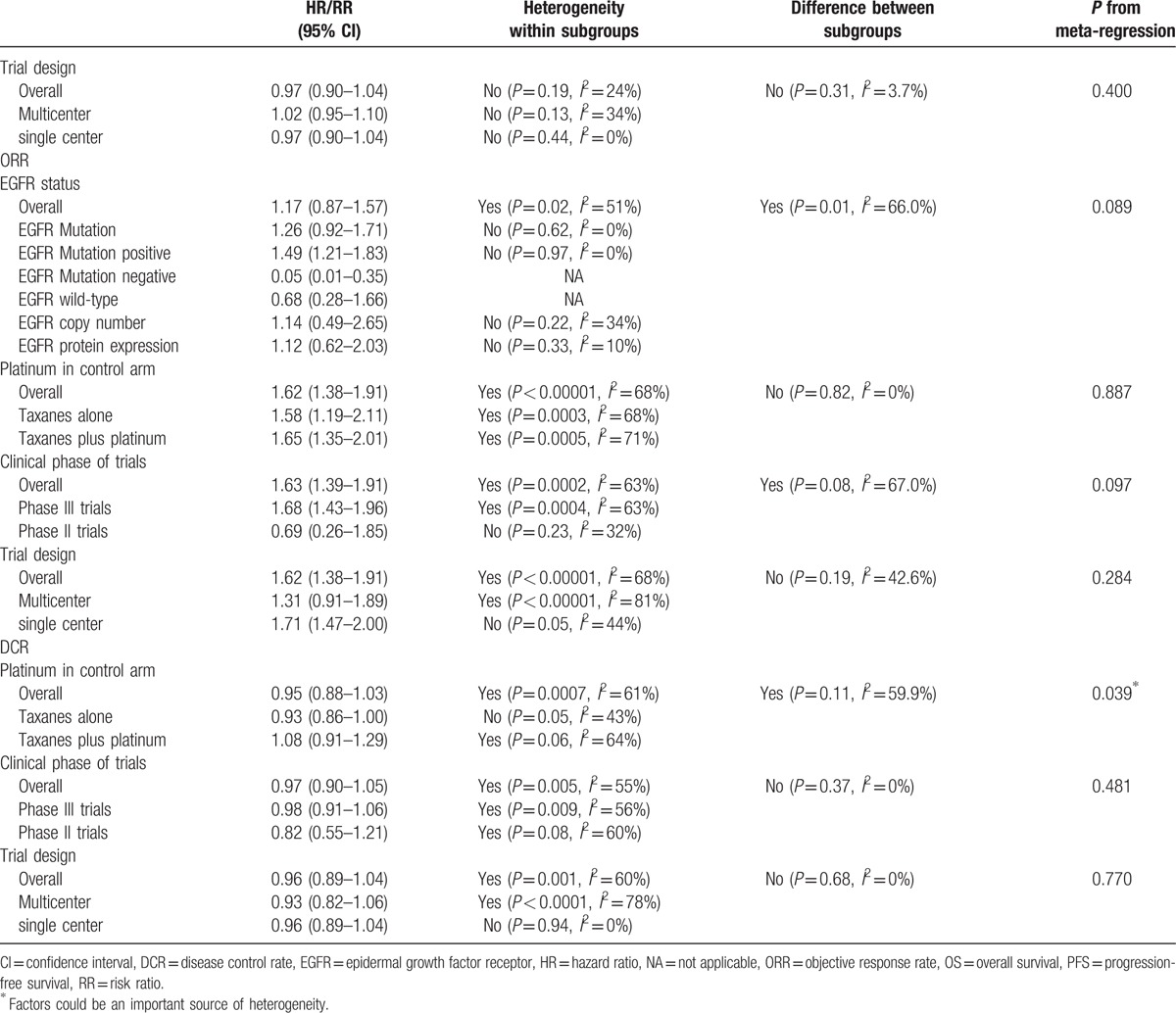
Summary of subgroup meta-analyses and meta-regression.

PFS: the 1st subgroup was performed on EGFR status, which result showed that comparing taxanes, EGFR-TKIs can significantly prolong PFS in patients with EGFR mutation (HR = 0.57, 95% CI: 0.43–0.76), EGFR mutation-positive (HR = 0.42, 95% CI: 0.27–0.65), and unknown EGFR mutation (HR = 0.68, 95% CI: 0.58–0.80). But EGFR-TKIs were inferior to taxanes in EGFR wild-type patients (HR = 1.32, 95% CI: 1.11–1.57) and EGFR mutation-negative (HR = 1.59, 95% CI: 0.50–5.02). There was no significant difference in EGFR copy number and EGFR protein expression. The 2nd subgroup grouped by platinum in control arm, the results demonstrated that EGFR-TKIs had an advantage over taxanes plus platinum (HR = 0.42, 95% CI: 0.27–0.65) and no advantage over taxanes alone (HR = 1.09, 95% CI: 0.98–1.20) in PFS. According to clinical phase of trials, the 3rd subgroup analysis result showed that the superiority of EGFR-TKIs over taxanes in phase III trials (HR = 0.77, 95% CI: 0.66–0.90). No difference was detected between 2 arms in phase II trials. The last subgroup under trial design, multicenter studies, and single center studies presented that the significant improvement of PFS was found in the EGFR-TKIs group compared with the taxanes groups (HR = 0.76, 95% CI: 0.64–0.89).

OS: As opposed to taxanes, EGFR-TKIs had a tendency to extend OS in patients with EGFR mutation (HR = 0.72, 95% CI: 0.40–1.29), EGFR mutation-positive (HR = 0.93, 95% CI: 0.74–1.18), and unknown EGFR mutation (HR = 0.75, 95% CI: 0.67–0.84); however, patients with EGFR mutation-negative (HR = 1.25, 95% CI: 0.97–1.61) was opposite. There were similar treatment effects between them in patients with EGFR wild-type, EGFR copy number, and EGFR protein expression. Six studies were grouped into taxanes plus platinum and 10 studies were grouped into taxanes alone, the result displayed no significant difference between EGFR-TKIs and taxanes in OS (HR = 1.00, 95% CI: 0.95–1.05). According to clinical phase of trials, which result showed there was no OS benefit for EGFR-TKIs over taxanes (HR = 0.99, 95% CI: 0.94–1.04). Multicenter had 10 studies and an additional 5 studies were single center, the overall result showed EGFR-TKIs had no significant difference in OS in NSCLC patients (HR = 0.97, 95% CI: 0.90–1.04).

ORR: The results indicated that there was benefit for EGFR-TKIs over taxanes in NSCLC patients with EGFR mutation (HR = 1.26, 95% CI: 0.92–1.71) and EGFR mutation-positive (HR = 1.49, 95% CI: 1.21–1.83), but patients with EGFR mutation-negative (HR = 0.05, 95% CI: 0.01–0.35) and EGFR wild-type (HR = 0.68, 95% CI: 0.28–1.66) were converse. EGFR-TKIs had equally therapy value to taxanes in EGFR copy number and EGFR protein expression patients. According to platinum in control arm (RR = 1.62, 95% CI: 1.38–1.91), clinical phase of trials (RR = 1.63, 95% CI: 1.39–1.91), and trial design (RR = 1.62, 95% CI: 1.38–1.91), we can make a conclusion that EGFR-TKIs had sustained clinical improvements over taxanes for patients in ORR.

DCR: After 3 subgroup analyses, the conclusion demonstrated whether paclitaxel or docetaxel, EGFR-TKIs cannot significantly improve DCR in NSCLC patients, the detail data were showed in Table 3 .

We also did meta-regression to find the source of heterogeneity. We found that grouping by platinum in control arm revealed differences in outcomes of PFS and DCR with *P* value less than 0.05. Moreover, EGFR status might have influenced heterogeneity in PFS (*P* = 0.039). Besides, grouping by clinical phase of trials, differences could be found in OS (*P* = 0.036).

### 3.5 Publication bias

**Figure 6:**
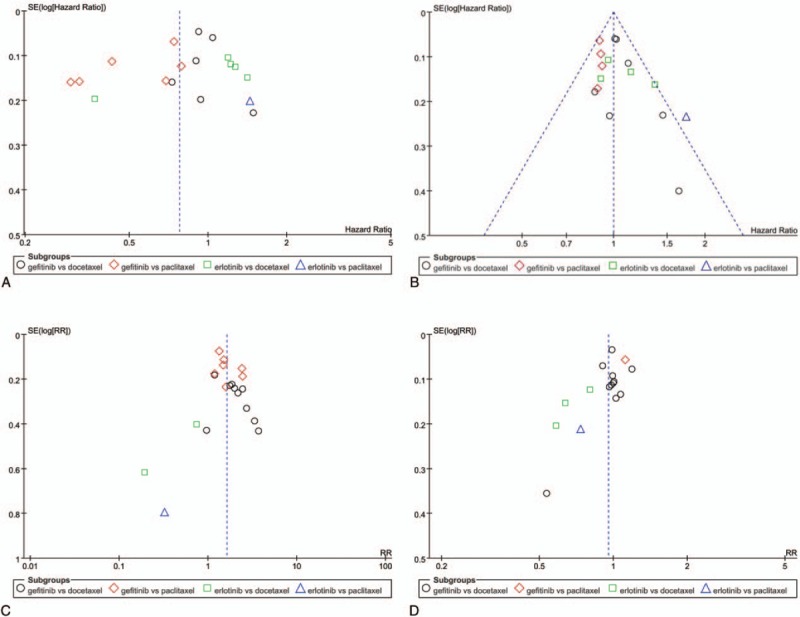
Funnel plot of comparison for PFS (A), OS (B), objective response rate (C), and disease control rate (D) between gefitinib and taxanes in NSCLC. NSCLC = nonsmall-cell lung cancer, OS = overall survival, PFS = progression-free survival.

We did the funnel plot according to PFS, OS, ORR, and DCR was shown in Fig. 6. The funnel plot showed asymmetry among our included studies, which proved the existence of publication bias.

## 4 Discussion

We carried out this meta-analysis to compare PFS, PFSR, OS, OSR, ORR, DCR, QoL, and AEs between EGFR-TKIs and taxanes. EGFR-TKIs can significantly prolong PFS and PFSR after therapy. The therapeutic effects of EGFR-TKIs were similar to taxanes in OS. Furthermore, taxanes were inferior to EGFR-TKIs in ORR. There was no significant difference between EGFR-TKIs and taxanes in DCR, while taxanes had a tendency to improve DCR. We found whether in FACT-L, LCS, or TOI, the results showed EGFR-TKIs surpassed taxanes in QoL with NSCLC patients. We found that comparing taxanes, NSCLC patients with EGFR mutation, EGFR mutation-positive, and unknown EGFR mutation can benefit from EGFR-TKIs on PFS, OS, and ORR. However, they cannot get beneficial treatment, who with EGFR wild-type and EGFR mutation-negative. There was no significant difference in EGFR copy number and EGFR protein expression. Thus, EGFR-TKIs are more suitable for patients with EGFR mutations and EGFR mutation-positive. Li et al^[46]^ made a relevant study, they also found that EGFR-TKIs were more efficient in EGFR mutations patients.

As per the analysis of heterogeneity, we did meta-regression and detail subgroup with EGFR status, platinum in control arm, clinical phase of trials, and trial design. First after EGFR status subgroup analyses, although the results showed PFS and ORR were different to drug groups, they had same tendency with drug groups. EGFR status on the effect of OS had difference on drug groups; hence, EGFR status might cause heterogeneity. Besides, we used platinum in control arm, clinical phase of trials, and trial design were operated in different groups, the results of PFS, OS, ORR, and DCR were equivalent to drug groups. However, analysis by meta-regression was found that platinum in control arm and clinical phase of trials were same to lead to heterogeneity, the detail results were showed in Table 3 . Subsequent analysis will be confirmed. In fact, small sample size may be one of the causes of heterogeneity.

There are also published meta-analysis, such as that by Zhao et al^[3]^ who compared the therapeutic values of gefitinib versus docetaxel, while we included more studies and outcomes. In fact, incorporating more clinical outcomes will bring more strong evidences in evaluation of efficacy and safety with NSCLC patients. Furthermore, we expanded the sample size and obtained consistent with their conclusions. To date, there has been no published meta-analysis comparing the efficacy and safety between EGFR-TKIs and taxanes. Pilkington et al^[47]^ published a systematic review of the clinical effectiveness of 1st-line chemotherapy, and they mentioned that, compared with paclitaxel and platinum, gefitinib had a statistically significant improvement in PFS, which was also consistent with the findings of our study, proving above-described results were trustworthy.

EGFR-TKIs could prolong PFS, improve ORR and QoL, yet they have many side-effects, such as rash and diarrhea.^[48,49]^ Taxanes also have several AEs: gastrointestinal reaction, alopecia, and hematological toxicity, particularly grade 3/4 leukopenia and neutropenia which tended to be more frequent after treatment with taxanes.^[50,51]^ In the meta-analysis of this study, except for diarrhea and rash, there was a slightly worse trend toward EGFR-TKIs compared with taxanes. EGFR-TKIs were superior to taxanes in rates of many AEs, such as all hematologic toxicity, myalgia, and pyrexia, etc. All of the data were listed in Table 2. It illustrated that the risk of AE rates was not increased when EGFR-TKIs instead of taxanes were applied for the treatment of NSCLC.

Holistic nursing care can improve the curable effects and significantly reduce adverse effects in the treatment of patients with hematological system disorders using high-dose dexamethasone pulse, and it deserves to be promoted to clinic.^[52]^ Auricular acupressure can significantly reduce the gastrointestinal side effects in lung cancer patients after chemotherapy, and be without any adverse reaction and high compliance.^[53]^ Meanwhile pantoprazole joint granisetron and methoxychlor Puan and dexamethasone prevent chemotherapy-induced gastrointestinal reactions with better efficacy, adverse reactions are mild, worthy of clinical application.^[54]^

Certainly this meta-analysis had several limitations need to be addressed. First most of included trials were allocated in Asian region (Table 1), which may cause the geographical limitations. Besides, due to limited or missing data about current trials, details such as gender, age, smoking, and cancer stage were unable to be analyzed. Moreover, not all of the patients in this study were serious, especially the performance status ≦2, which may be proved that the basic level was mixed. Although anticancer drugs have been used widely in NSCLC, related randomized clinical trials appear to be limited. Furthermore, the different outcome assessment times could lead to the existence of publication bias. Positive results are easy to be published, negative results with several AEs are not likely to be viewed. Finally the quality of included studies were variable, although most of them with acceptable quality, high-quality, well-level, and large-scale double-blind RCTs are needed for further research. Considering the limitations above, further studies were warranted to complete the information and the results of this research must be interpreted with caution.

In terms of PFS, PFSR, ORR, QoL, and AEs, EGFR-TKIs were superior to taxanes in NSCLC patients from the present meta-analysis study, particularly who were with EGFR mutation-positive. There were no differences between EGFR-TKIs and taxanes in OS, OSR, and DCR. From a clinical perspective, no matter the efficacy or the toxicity, EGFR-TKIs are significant difference potential and valuable choices in the treatment of NSCLC. Certainly we need more high-quality and large-scale RCTs for further research.

## Supplementary Material

Supplemental Digital Content
