# Supplementary material for: EGFR-TKIs versus taxanes agents in therapy for nonsmall-cell lung cancer patients: A PRISMA-compliant systematic review with meta-analysis and meta-regression
Source: Medicine (Baltimore). 2016 Dec 16;95(50):e5601. doi: 10.1097/MD.0000000000005601 (PMC5268044; doi:10.1097/MD.0000000000005601)
Supplement: Supplemental Digital Content [file md-95-e5601-s001.doc]

Figure S1. Risk of bias in the included studies.


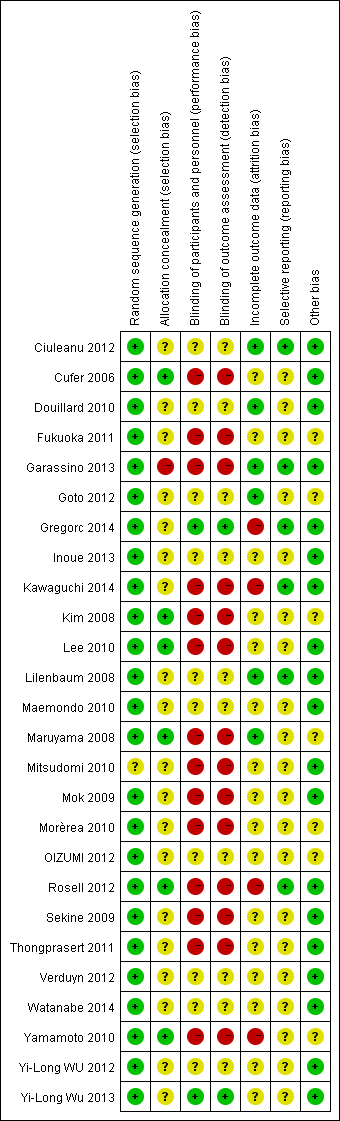


**Table S1.** Search strategies

**Search strategies for PubMed**

#1. (("EGFR-TKIs"[MeSH Terms] OR "EGFR-TKIs"[All Fields]) AND ("taxanes"[MeSH Terms] OR "taxanes"[All Fields])) AND Randomized Controlled Trial[ptyp]

#2. (("EGFR-TKIs"[MeSH Terms] OR "EGFR-TKIs"[All Fields]) AND ("taxanes"[MeSH Terms] OR "taxanes"[All Fields])) AND Clinical Study[ptyp]

#3. (("gefitinib"[MeSH Terms] OR "gefitinib"[All Fields]) AND ("docetaxel"[MeSH Terms] OR "docetaxel"[All Fields] AND Randomized Controlled Trial[ptyp]

#4. (("gefitinib"[MeSH Terms] OR "gefitinib"[All Fields]) AND ("docetaxel"[MeSH Terms] OR "docetaxel"[All Fields])) AND Clinical Study[ptyp]

#5. (("gefitinib"[MeSH Terms] OR "gefitinib"[All Fields]) AND ("paclitaxel"[MeSH Terms] OR "paclitaxel"[All Fields] AND Randomized Controlled Trial[ptyp]

#6. (("gefitinib"[MeSH Terms] OR "gefitinib"[All Fields]) AND ("paclitaxel"[MeSH Terms] OR "paclitaxel"[All Fields])) AND Clinical Study[ptyp]

#7. (("erlotinib"[MeSH Terms] OR "erlotinib"[All Fields]) AND ("docetaxel"[MeSH Terms] OR "docetaxel"[All Fields])) AND Randomized Controlled Trial[ptyp]

#8. (("erlotinib"[MeSH Terms] OR "erlotinib"[All Fields]) AND ("docetaxel"[MeSH Terms] OR "docetaxel"[All Fields])) AND Clinical Study[ptyp]

#9. (("erlotinib"[MeSH Terms] OR "erlotinib"[All Fields]) AND ("paclitaxel"[MeSH Terms] OR "paclitaxel"[All Fields])) AND Randomized Controlled Trial[ptyp]

#10. (("erlotinib"[MeSH Terms] OR "erlotinib"[All Fields]) AND ("paclitaxel"[MeSH Terms] OR "paclitaxel"[All Fields])) AND Clinical Study[ptyp]

#11. (("afatinib"[MeSH Terms] OR "afatinib"[All Fields]) AND ("docetaxel"[MeSH Terms] OR "docetaxel"[All Fields])) AND Randomized Controlled Trial[ptyp]

#12. (("afatinib"[MeSH Terms] OR "afatinib"[All Fields]) AND ("docetaxel"[MeSH Terms] OR "docetaxel"[All Fields])) AND Clinical Study[ptyp]

#13. (("afatinib"[MeSH Terms] OR "afatinib"[All Fields]) AND ("paclitaxel"[MeSH Terms] OR "paclitaxel"[All Fields])) AND Randomized Controlled Trial[ptyp]

#14. (("afatinib"[MeSH Terms] OR "afatinib"[All Fields]) AND ("paclitaxel"[MeSH Terms] OR "paclitaxel"[All Fields])) AND Clinical Study[ptyp]

#15. (("icotinib"[MeSH Terms] OR "icotinib"[All Fields]) AND ("docetaxel"[MeSH Terms] OR "docetaxel"[All Fields])) AND Randomized Controlled Trial[ptyp]

#16. (("icotinib"[MeSH Terms] OR "icotinib"[All Fields]) AND ("docetaxel"[MeSH Terms] OR "docetaxel"[All Fields])) AND Clinical Study[ptyp]

#17. (("icotinib"[MeSH Terms] OR "icotinib"[All Fields]) AND ("paclitaxel"[MeSH Terms] OR "paclitaxel"[All Fields])) AND Randomized Controlled Trial[ptyp]

#18. (("icotinib"[MeSH Terms] OR "icotinib"[All Fields]) AND ("paclitaxel"[MeSH Terms] OR "paclitaxel"[All Fields])) AND Clinical Study[ptyp]

**Search strategies for EMbase**

#1. 'EGFR-TKIs'/exp OR EGFR-TKIs AND 'taxanes'/exp OR taxanes AND Randomized controlled trial OR Clinical trail

#2. 'gefitinib'/exp OR gefitinib AND 'docetaxel'/exp OR docetaxel AND Randomized controlled trial OR Clinical trail

#3. 'gefitinib'/exp OR gefitinib AND 'paclitaxel'/exp OR paclitaxel AND Randomized controlled trial OR Clinical trail

#4. 'erlotinib'/exp OR erlotinib AND 'docetaxel'/exp OR docetaxel AND Randomized controlled trial OR Clinical trail

#5. 'erlotinib'/exp OR erlotinib AND 'paclitaxel'/exp OR paclitaxel AND Randomized controlled trial OR Clinical trail

#6. 'afatinib'/exp OR afatinib AND 'docetaxel'/exp OR docetaxel AND Randomized controlled trial OR Clinical trail

#7. 'afatinib'/exp OR afatinib AND 'paclitaxel'/exp OR paclitaxel AND Randomized controlled trial OR Clinical trail

#8. 'icotinib'/exp OR icotinib AND 'docetaxel'/exp OR docetaxel AND Randomized controlled trial OR Clinical trail

#9. 'icotinib'/exp OR icotinib AND 'paclitaxel'/exp OR paclitaxel AND Randomized controlled trial OR Clinical trail

**Search strategies for Cochrane library**

#1.EGFR-TKIs and taxanes; gefitinib and docetaxel or paclitaxel; erlotinib and docetaxel or paclitaxel; afatinib and docetaxel or paclitaxel; icotinib and docetaxel or paclitaxel;

#2. MeSH descriptor

**Table S2.** The quality of studies assessed by Newcastle-Ottawa Scale.

**S2 Table Risk of Bias Assessments**

Risk of Bias Assessment using the Newcastle-Ottawa Scale for Case-control Studies

| Low Risk of Bias |  |
| --- | --- |
| Intermediate or Unknown Risk of Bias |  |
| High Risk of Bias |  |

| Study | Is the case definition adequate | Representativeness of the cases | Selection of Controls | Definition of Controls | Comparability of cases and controls (/2) | Ascertainment of exposure(/2) | Same method of ascertainment for cases and controls | Non-Response rate | Overall rating and TOTAL SCORE / 10 |
| --- | --- | --- | --- | --- | --- | --- | --- | --- | --- |
| Cufer 2006 | 1 | 1 | 0 | 0 | 2 | 1 | 1 | 1 | 7 |
| Kim 2008 | 1 | 1 | 0 | 0 | 2 | 1 | 1 | 1 | 7 |
| Maruyama 2008 | 1 | 1 | 0 | 1 | 2 | 1 | 1 | 1 | 8 |
| Sekine 2009 | 1 | 1 | 0 | 1 | 1 | 1 | 1 | 1 | 7 |
| Douillard 2010 | 1 | 1 | 0 | 1 | 2 | 1 | 1 | 1 | 8 |
| Lee 2010 | 1 | 1 | 0 | 0 | 2 | 1 | 1 | 1 | 7 |
| Morèrea 2010 | 1 | 1 | 0 | 1 | 2 | 1 | 1 | 1 | 8 |
| Mitsudomi 2010 | 1 | 1 | 0 | 0 | 2 | 1 | 1 | 1 | 7 |
| Yamamoto 2010 | 1 | 1 | 0 | 1 | 2 | 1 | 1 | 1 | 8 |
| Ciuleanu 2012 | 1 | 1 | 0 | 0 | 2 | 1 | 1 | 1 | 7 |
| Rosell 2012 | 1 | 1 | 0 | 1 | 2 | 1 | 1 | 1 | 8 |
| Garassino 2013 | 1 | 1 | 0 | 1 | 2 | 1 | 1 | 1 | 8 |
| Gregorc 2014 | 1 | 1 | 0 | 0 | 2 | 2 | 1 | 1 | 8 |
| Kawaguchi 2014 | 1 | 1 | 0 | 0 | 2 | 1 | 1 | 1 | 7 |
| Mok 2009 | 1 | 1 | 0 | 0 | 2 | 1 | 1 | 1 | 7 |
| Maemondo 2010 | 1 | 1 | 0 | 0 | 2 | 1 | 1 | 1 | 7 |
| Fukuoka 2011 | 1 | 1 | 0 | 1 | 2 | 1 | 1 | 1 | 8 |
| Thongprasert 2011 | 1 | 0 | 0 | 0 | 2 | 1 | 1 | 1 | 6 |
| Goto 2012 | 1 | 1 | 0 | 1 | 2 | 1 | 1 | 1 | 8 |
| OIZUMI 2012 | 1 | 1 | 0 | 1 | 2 | 1 | 1 | 1 | 8 |
| Verduyn 2012 | 1 | 0 | 0 | 1 | 1 | 1 | 1 | 1 | 6 |
| Yi-Long WU 2012 | 1 | 1 | 0 | 0 | 2 | 1 | 1 | 1 | 7 |
| Inoue 2013 | 1 | 1 | 0 | 0 | 2 | 1 | 1 | 1 | 7 |
| Yi-Long Wu 2013 | 1 | 1 | 0 | 1 | 1 | 2 | 1 | 1 | 8 |
| Watanabe 2014 | 1 | 1 | 0 | 1 | 2 | 0 | 1 | 1 | 7 |
| Lilenbaum 2008 | 1 | 1 | 0 | 0 | 2 | 1 | 1 | 1 | 7 |
